# Supplementary material for: Galectin-3 captures interferon-gamma in the tumor matrix reducing chemokine gradient production and T-cell tumor infiltration
Source: Nat Commun. 2017 Oct 6;8:793. doi: 10.1038/s41467-017-00925-6 (PMC5630615; doi:10.1038/s41467-017-00925-6)
Supplement: Supplementary file 1 — Supplementary Information [file 41467_2017_925_MOESM1_ESM.pdf]

Description of Supplementary Files

File Name: Supplementary Information
Description: Supplementary Figures, Supplementary Table and Supplementary Methods

File Name: Peer Review File

Supplementary Methods

CCL5 and CCL1 binding to galectin-3-beads

Purified recombinant human glycosylated CCL5 (Origene; TP303799) was produced by HEK cells. Professor J. Van Snick (Ludwig Institute for Cancer Research, Brussels) kindly provided purified recombinant human glycosylated CCL1. The binding was performed as previously described for IFNγ. CCL5 in the solution was measured by Bioplex (Biorad). CCL1 was measured using a cell-reporter system (*described in J. van Snick et al. 1996 J Immunol*).

CXCL10 and PD-L1 mRNA induction in vitro

Tumor cells were seeded in P6 wells. Three days later, confluent cells were treated for 4 h with 50 ng ml^{-1} IFNγ, 5 mM LacNAc, and/or 5 μg ml^{-1} neutralizing anti-human IFNγR1 mouse IgG1 antibody (GIR-208 clone; R&D MAB6732). Cells were lysed and RNA was extracted as explained in the main experimental methods.

Reverse transcription and Real Time PCR

RNA amount, purity and integrity were measured by spectrophotometry (Nanodrop 2000) and by the 2100 Bioanalyzer (Agilent Technologies). RIN values for the *in vitro* and *in vivo* samples were 9-10 whereas the RIN values for the *ex vivo* samples were 6.5-9. Genomic DNA amplification, PCR efficiency and no template controls were checked for all the oligos-probe sets, following MIQE guidelines (Bustin et al., 2009). Reverse transcription was performed with 2 μg of total RNA with PrimeScript RT Reagent Kit (Takara; RR037A) and no random 6 mers were added. RT-PCR reactions were performed in technical triplicates. All samples were analyzed using several reference genes getting similar results. HPRT-1 was the most reproducible reference gene for the tumor samples. Human/murine β-Actin was selected as a reference gene for analyzing the hCD3ε/hCD8α content in tumor and spleen samples. Noteworthy, HPRT-1 oligos-probe were only amplifying human cDNA and not murine cDNA. RT-PCR data were analyzed manually using the comparative method.

Supplementary Table 1. Oligos and probes used for RT-PCR analysis.

Target	Amplicon length	Oligos and probe for RT-PCR (sense/antisense/probe written 5' to 3')
ACTB (murine/human)		CTC TGG CTC CTA GCA CCA TGA AG<br>GCT GGA AGG TGG ACA GTG AG<br>ATC GGT GGC TCC ATC CTG GC
CD3E	159	TGA GGG CAA GAG TGT GTG AG (exon 6-7)<br>CCG CTC CTC GTG TCA CAG (exon 7)<br>CAC CGA CAT CAC ATC CAT CTC CAT GCA G
CD8A	60	CCT GAG CAA CTC CAT CAT GT (exon 2)<br>GGC TTC GCT GGC AGG A (exon 3)<br>CTT CAG CCA CTT CGT GCC GGT
CXCL9	60	Hs00171065_m1 (Life Technologies)
CXCL10	74	Hs04421396-g1 (Life Technologies)
HPRT-1	100	Hs99999909_m1 (Life Technologies)
LGALS3	64	Hs00173587_m1 (Life Technologies)
PD-L1	79	GGA GAT TAG ATC CTG AGG AAA ACC A<br>CTT TCA TTT GGA GGA TGT GCC<br>AGC TGA ATT GGT CAT CCC AGA ACT ACC TC
TBP1	91	Hs00427620_m1 (Life Technologies)

All probes were coupled to FAM-TAMRA, except the ones from Life Technologies that were coupled to FAM-MGB

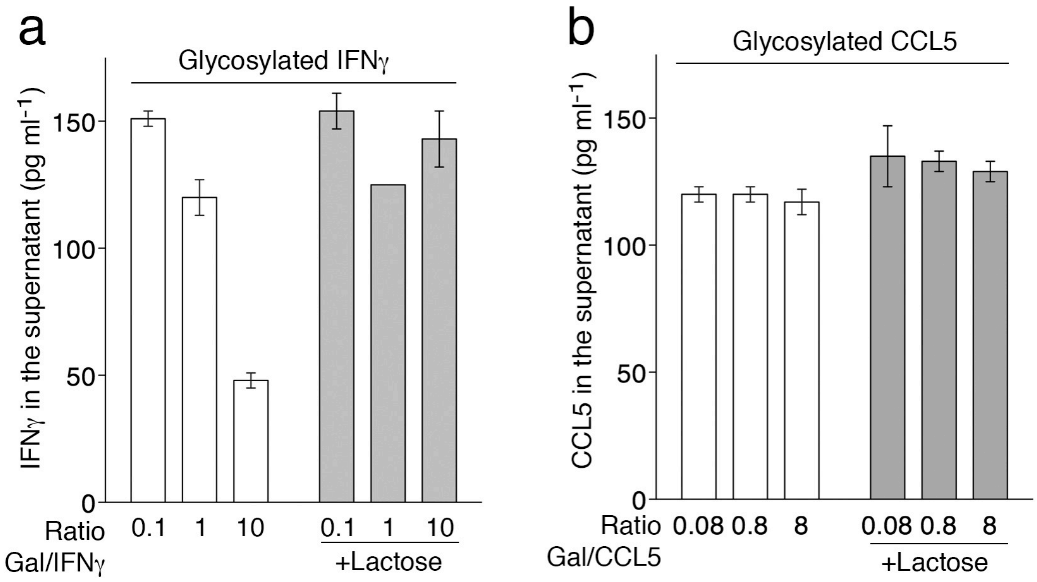

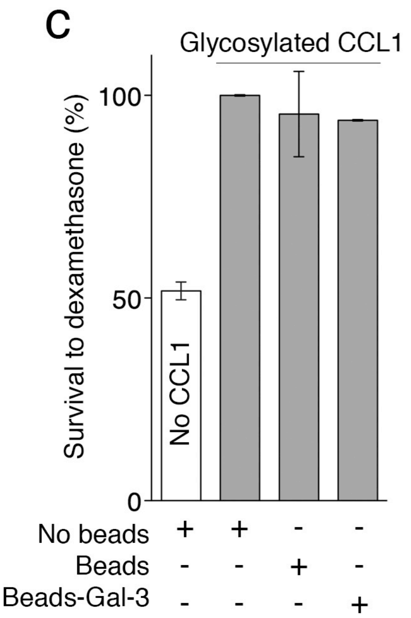

d

Glycosylation sites				Captured by Gal-3 beads	
Type	Number (predicted)	Number (published)		Recombinant protein	Proteins in synovial fluid (capture%)
O-glyc	1	1	IL-2	Not tested	37 %
N-glyc	1	0	IL-10	Not tested	40 %
N-glyc	4	1	IL-12	Yes	37 %
N-glyc	1	0	IL-15	Not tested	19 %
N-glyc	2	2	IFNγ	Yes	40 %
Unglyc	0	0	IFNγ	No	Not relevant
N-glyc	1	0	CCL1	No	Not tested
N-glyc	1	0	CCL2	Not tested	2 %
O-glyc	2	2	CCL5	No	0 %

Supplementary Figure 1. Binding of galectin-3 to glycosylated cytokines and chemokines. Ratio refers to molar ratio. a) Human glycosylated IFNγ produced by HEK cells was measured by ELISA in the supernatant after incubation with galectin-3-coated beads in the presence or absence of 100 mM lactose. One representative experiment of four performed in duplicates. b) O-glycosylated CCL5 measured by bioplex assay in the supernatant after incubation with galectin-3-coated beads in the presence or absence of 100 mM lactose. One representative experiment of 3 performed in duplicates. c) Percentage of BW5147C2 cells that survive to dexamethasone (200 ng ml^{-1}) in the absence or presence of glycosylated CCL1 after incubation with beads. One representative experiment of 3 performed in triplicates. d) Table summarizing the cytokines that were analyzed for galectin-3 binding using galectin-3-coated beads. Glycosylation sites data were taken from Uniprot database (www.uniprot.org).

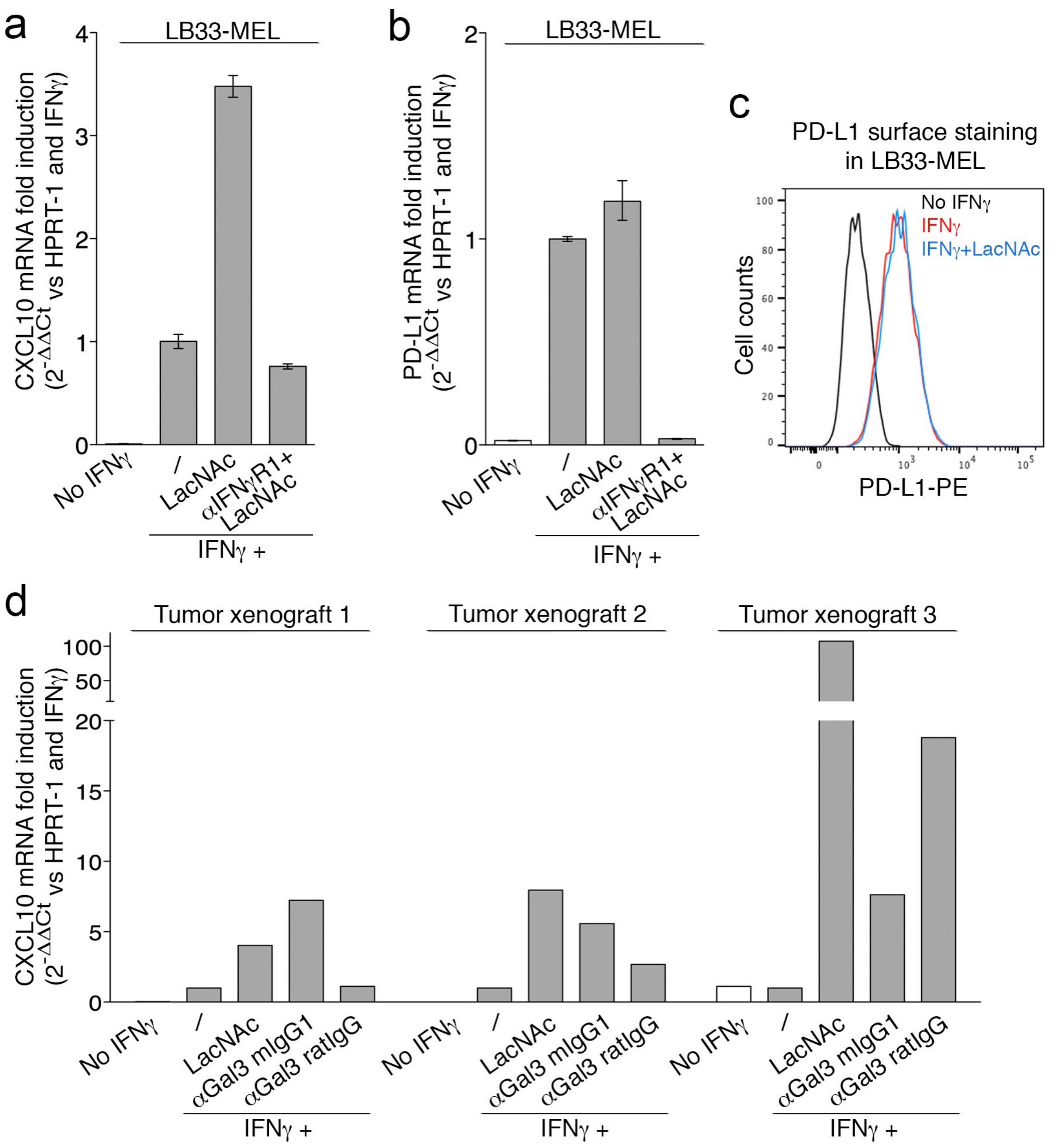

Supplementary Figure 2. IFNγ-induced chemokine expression is enhanced by galectin antagonists in human tumor cell lines and xenografts. mRNA fold induction values were calculated using HPRT-1 as reference gene and with respect to IFNγ−treated condition ($2^{-\Delta\Delta Ct}$). a) CXCL10 mRNA fold induction in LB33-MEL cell line incubated for 4 h with IFNγ (50 ng ml^{-1}), LacNAc (5 mM) or anti-IFNγR1 antibody (5 µg ml^{-1}). b) PD-L1 mRNA fold induction in LB33-MEL cell line incubated for 4 h with IFNγ (50 ng ml^{-1}), LacNAc (5 mM) or anti-IFNγR1 antibody (5 µg ml^{-1}). c) PD-L1 protein level was analyzed by flow cytometry three days after treatment with IFNγ (50 ng ml^{-1}) alone or combined with LacNAc (5 mM). d) CXCL10 mRNA fold induction in LB33-MEL xenografts treated ex vivo with IFNγ (50 ng ml^{-1}), LacNAc (10 mM) or antibodies (10 µg ml^{-1}). Three representative tumors out of 8 are shown.

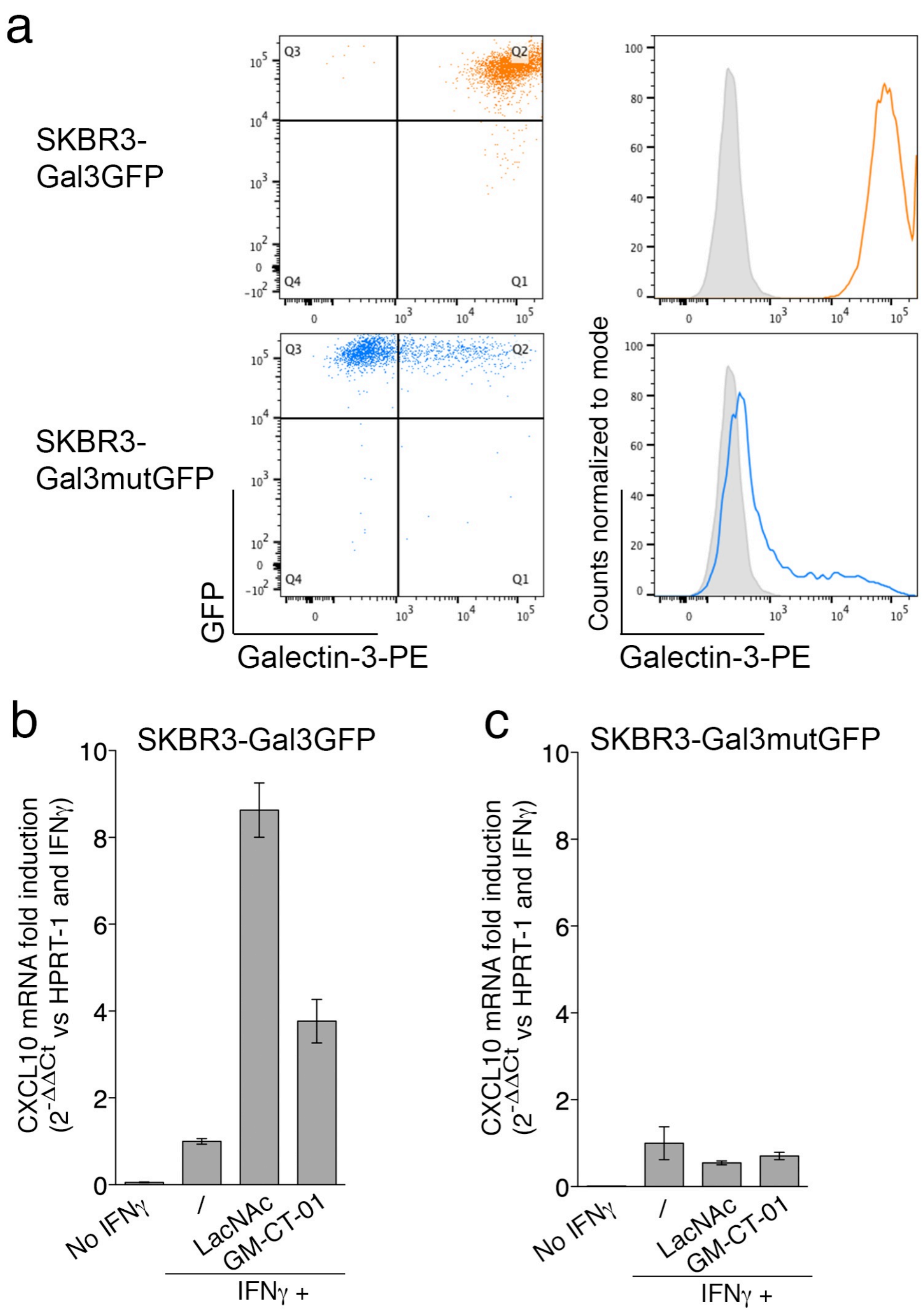

Supplementary Figure 3. Tumors expressing a mutated galectin-3 do not increase IFNγ-induced CXCL10 upon treatment with galectin antagonists. a) Galectin-3 surface staining for SKBR3-Gal3GFP (in orange) and SKBR3-Gal3mutGFP (in blue). Isotype control staining is shown as a grey histogram. Both cell lines have similar levels of GFP but different levels of surface galectin-3, as only galectin-3-GFP binds efficiently glycan motifs on the cells surface. b) CXCL10 mRNA fold induction for SKBR3-Gal3GFP xenografts treated ex vivo with IFNγ (50 ng ml^{-1}), LacNAc (5 mM), or GM-CT-01 (100 µg ml^{-1}). Mean ± SEM of one experiment pooling pieces from 6 tumors. c) CXCL10 mRNA fold induction for SKBR3-Gal3mutGFP xenografts treated ex vivo with IFNγ (50 ng ml^{-1}), LacNAc (5 mM) or GM-CT-01 (100 µg ml^{-1}). Mean ± SEM of one experiment pooling pieces from 8 tumors.

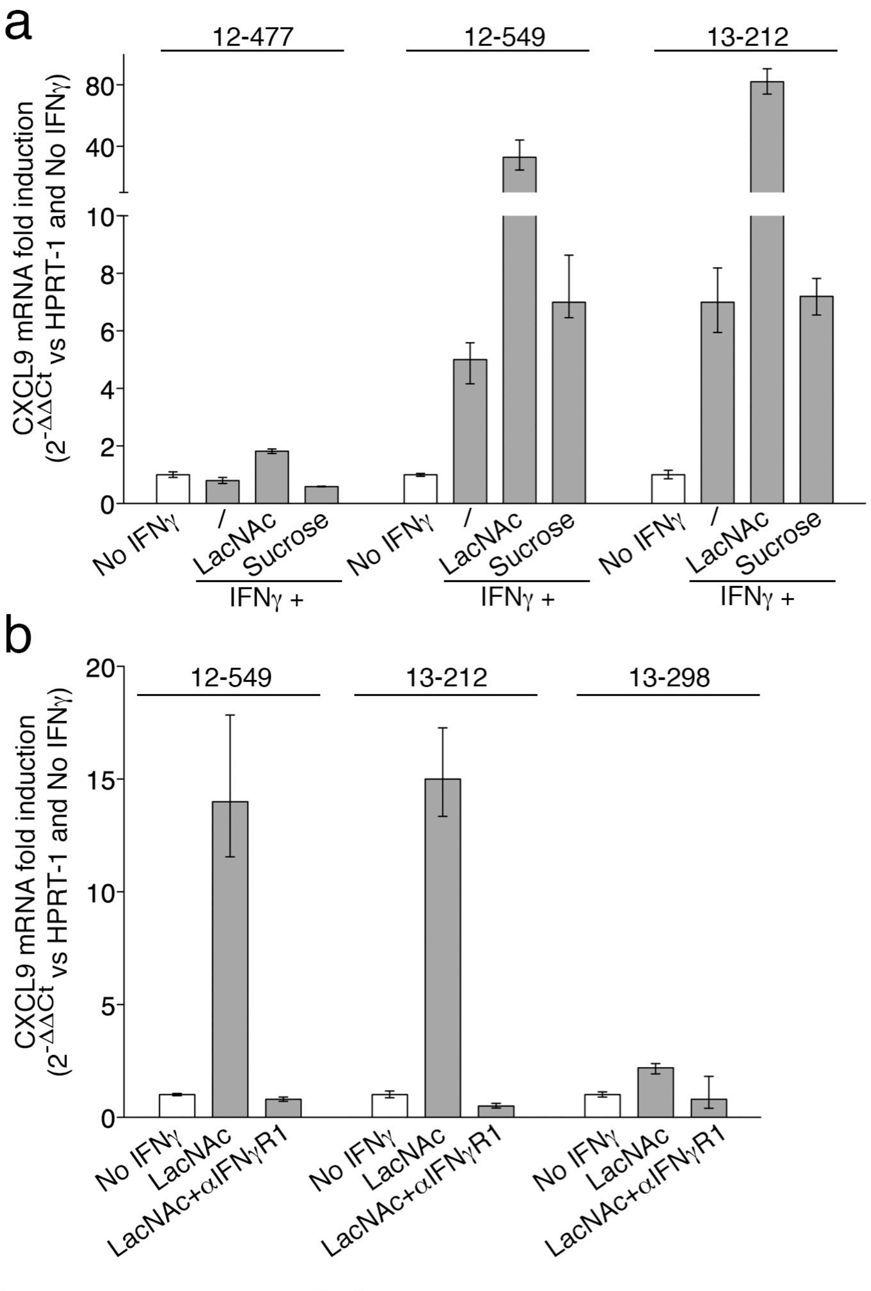

Supplementary Figure 4. CXCL9 expression in human tumor biopsies treated ex vivo with galectin antagonists. Human tumor biopsies were treated for 6 h ex vivo (hIFNγ 50 ng ml^{-1}, LacNAc 10 mM, sucrose 10 mM, anti-IFNγR-1 antibody 5 µg ml^{-1}). See Table 1 for the complete data. a) Examples showing the increased CXCL9 induction observed when biopsies were treated with IFNγ and LacNAc. b) Examples showing the effect of LacNAc treatment alone.

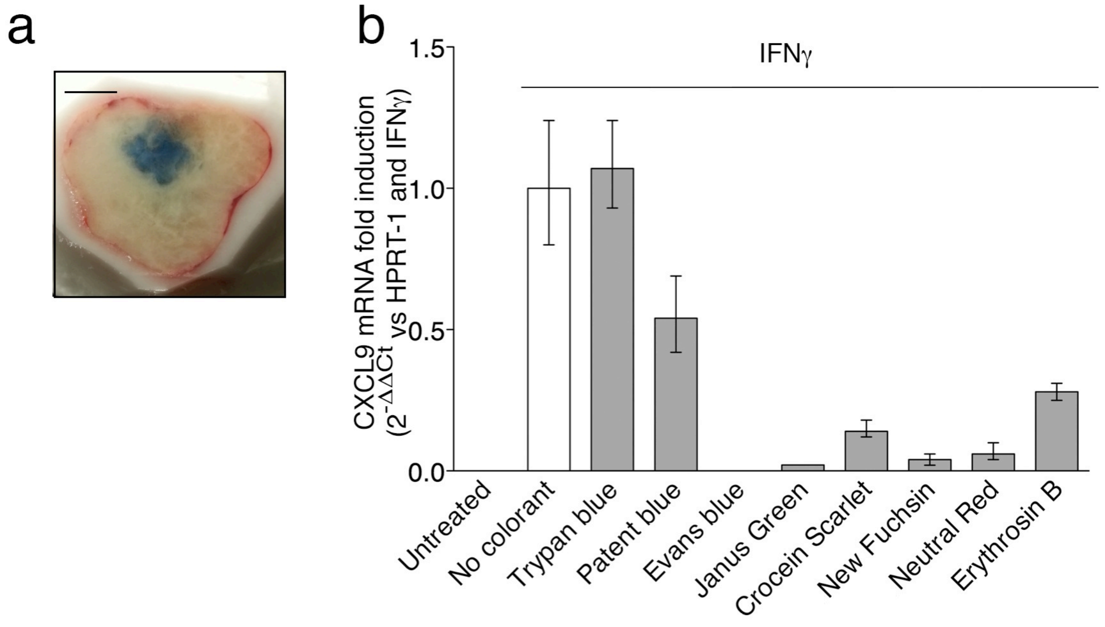

Supplementary Figure 5. Influence of colorants on CXCL9 expression in human tumor xenografts in vivo. a) Section of a tumor injected in its center with IFNγ in PBS 0.2% trypan blue. One day after, the tumor was extracted and frozen in OCT. Bar represents 5 mm. b) CXCL9 induction by IFNγ injected together with different colorants (trypan blue was used at 0.2 %, patent blue, crocein scarlet, new fuchsin, neutral red and evans blue at 0.15%, and janus green and erythrosin B at 0.08%). Only trypan blue did not affect IFNγ-induced CXCL9 expression.

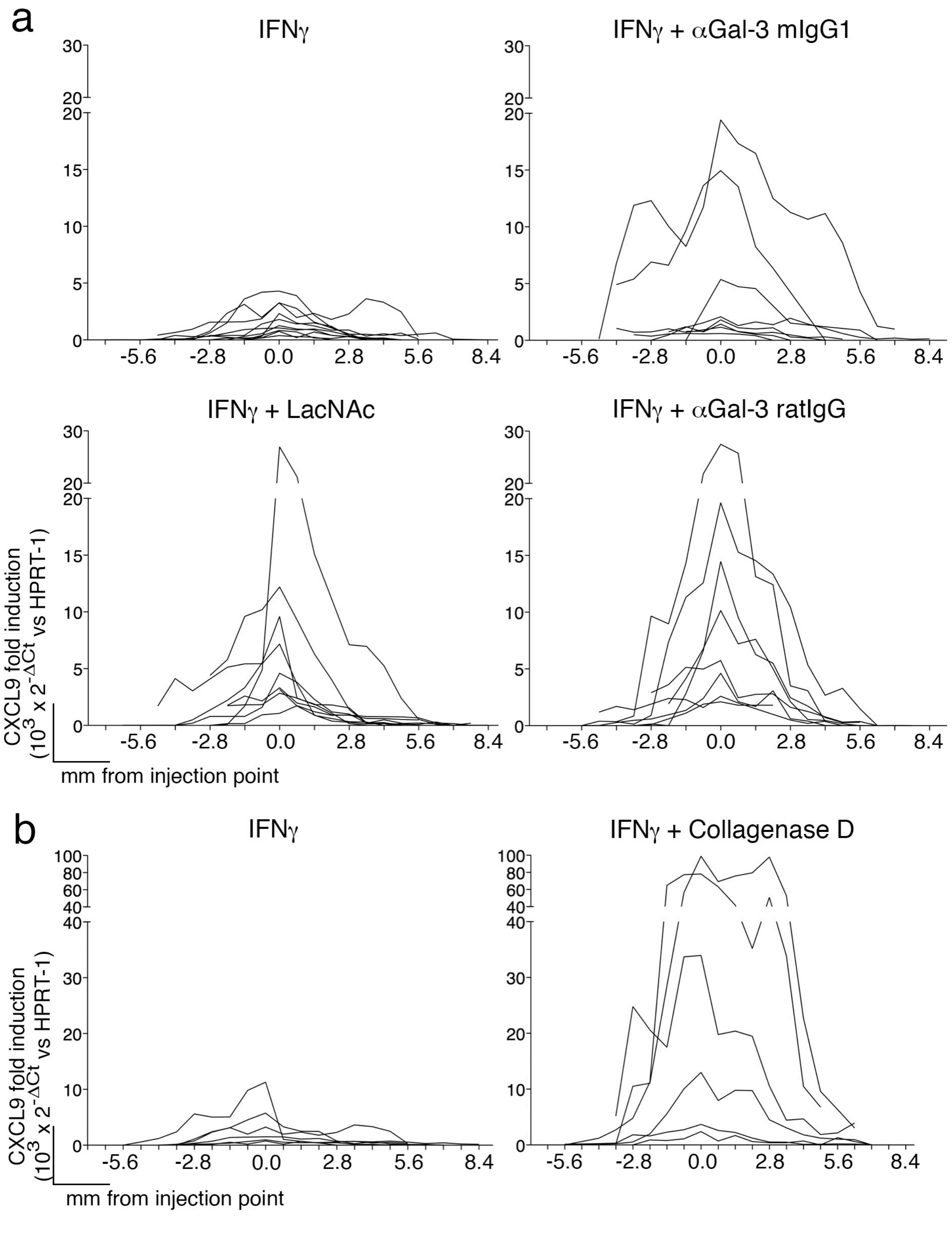

Supplementary Figure 6. Spreading of IFNγ signal in tumor xenografts treated in vivo with galectin antagonists or collagenase D. a-b) CXCL9 fold induction along the tumor sections in mice treated with IFNγ alone (50 ng per tumor) or together with LacNAc (0.1 µmol per tumor), antibodies (100 ng per tumor), or collagenase D (2.5 µg per tumor). Each line represents CXCL9 mRNA fold induction along the sections of one independent tumor. CXCL9 values were calculated using HPRT-1 as reference gene (10^3x $2^{-\Delta Ct}$). a) n=8 mice per group b) n=6 mice per group.

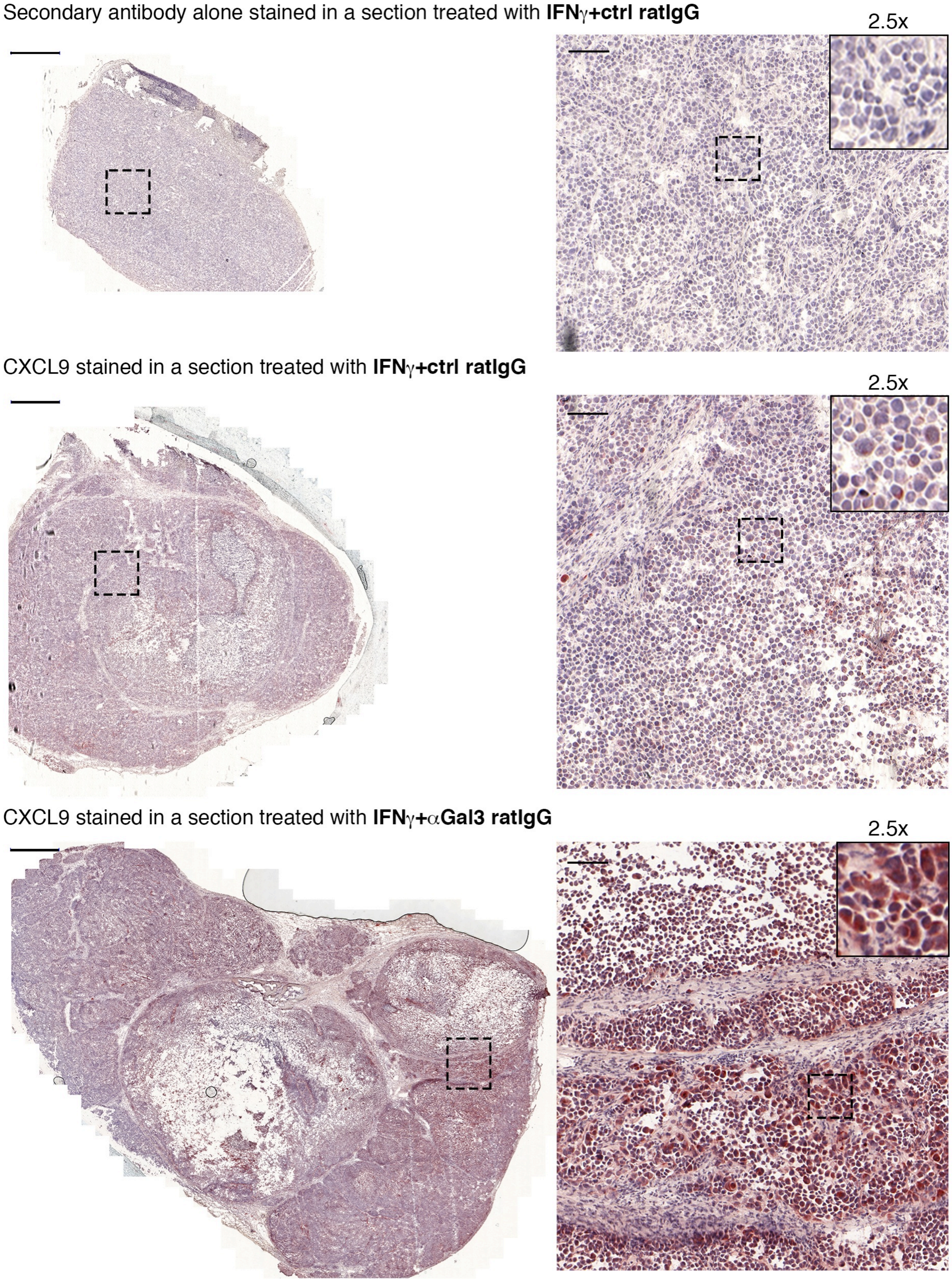

Supplementary Figure 7. CXCL9 staining of human tumor xenografts treated in vivo with IFNγ and galectin antagonists. Tumors treated with IFNγ and either a control isotype antibody or an anti-galectin3 ratIgG antibody, were stained for CXCL9 (in red), and counterstained with hematoxylin (in blue). The first panel shows the background when the tumor section was only stained with the secondary antibody. Several successive magnifications of the areas depicted with dotted squares are shown. Bars in the first column represent 1000 μm, and those in the second column 100 μm.

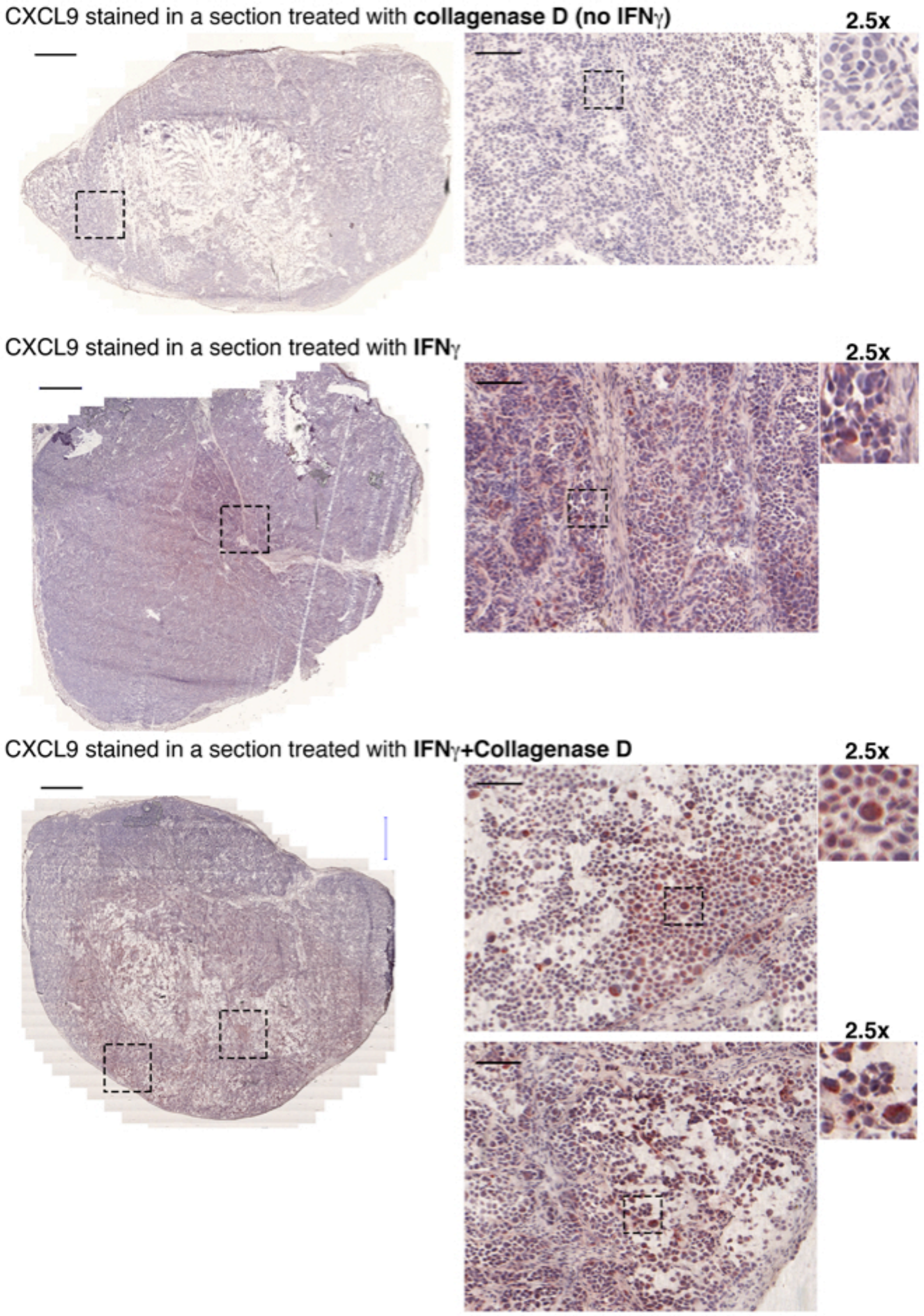

Supplementary Figure 8. CXCL9 staining of human tumor xenografts treated in vivo with IFNγ and collagenase D. Tumors treated with collagenase D, IFNγ, or IFNγ and collagenase D were stained for CXCL9 (in red) and counterstained with hematoxylin (in blue). Several successive magnifications of the areas depicted with dotted squares are shown. Bars in the first column represent 1000 μm and in the second column 100 μm. Matrix degradation is observed in the center of the slices treated with collagenase D alone or in combination with IFNγ. Collagen represents 15% of the tumor slice (average from 26 sections).

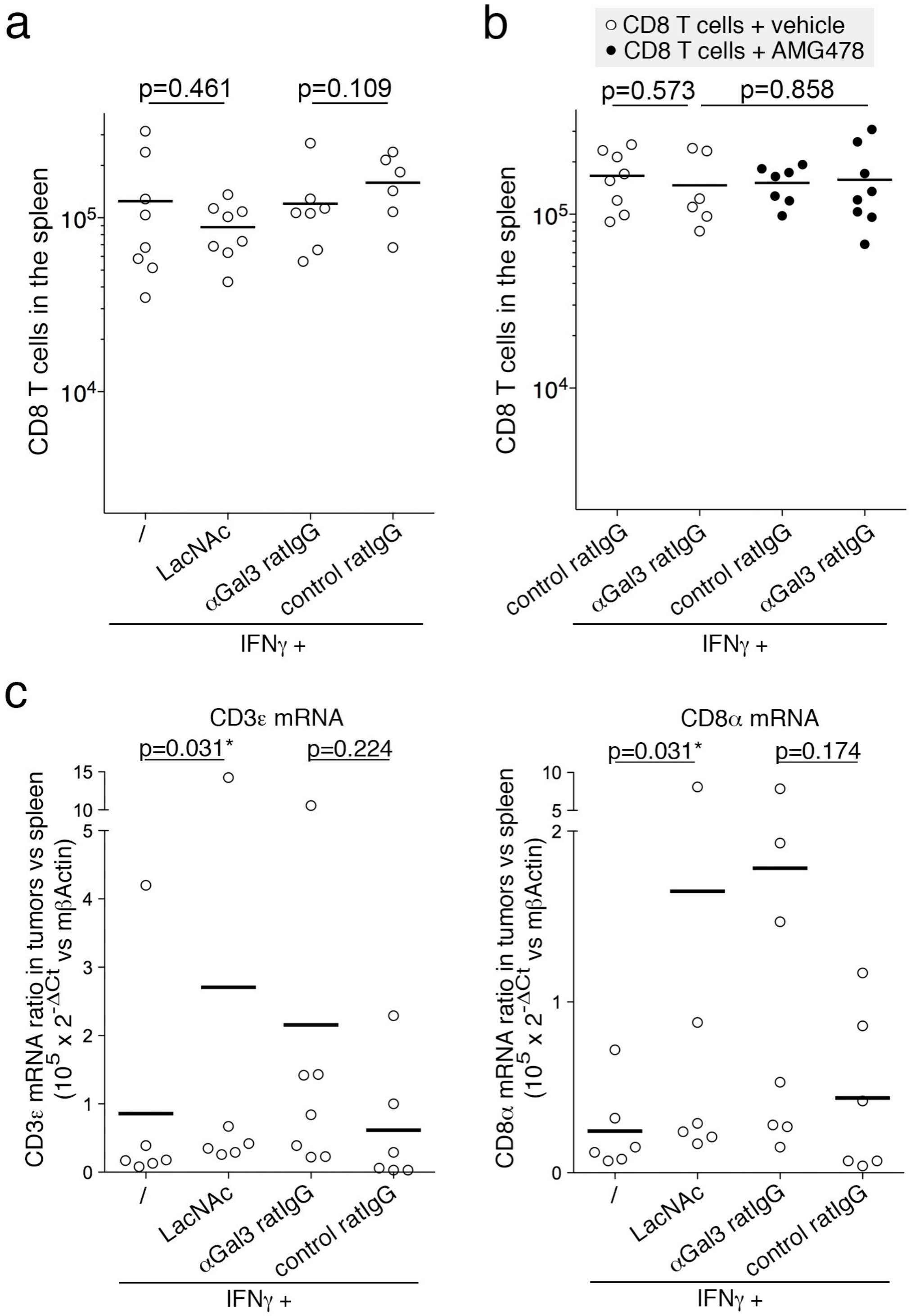

Supplementary Figure 9. Galectins antagonists improve tumor T cell infiltration. a - b) Graphs showing the absolute number of CD8 T cells found in the spleens (each point represents one mouse). Lines represent the mean for each treatment. Wilcoxon matched-pairs test with Dunn's Multiple Comparison Correction Test. P values are shown on the graphs. c) CD3ε and CD8α mRNA ratio of each tumor sample by its corresponding spleen. Lines represent the mean for each treatment. Wilcoxon matched-pairs test with Dunn's Multiple Comparison Correction Test. P values are shown on the graphs.

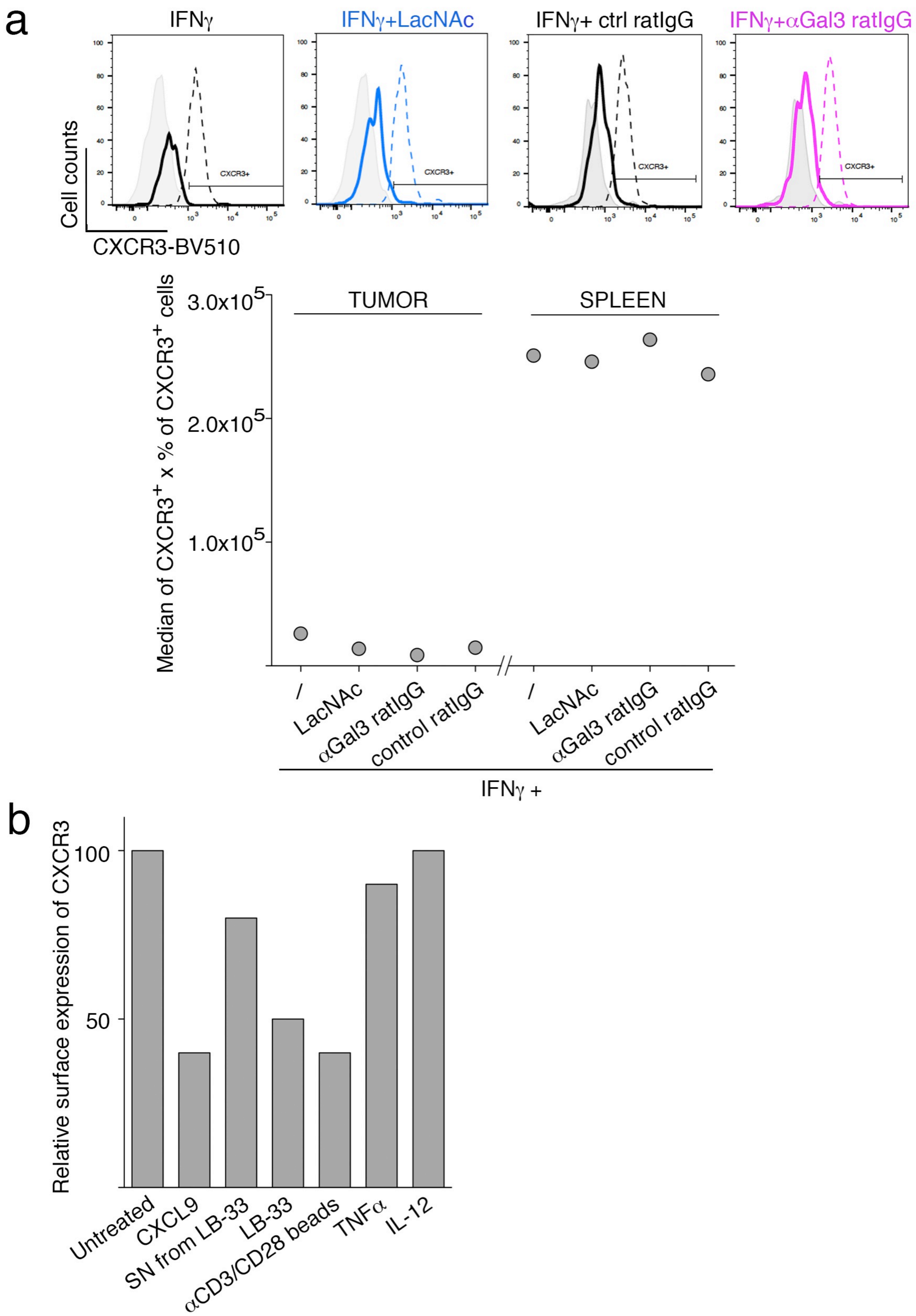

Supplementary Figure 10. CXCR3 downregulation on αMUM-3 CD8$^+$ T cell surface. a) Representative histograms showing CXCR3 surface staining for CD8$^+$ T cells found in tumors (bold lines) or in spleens (dotted lines). The graph below represents the mean surface CXCR3 staining for CD8$^+$ T cells obtained from tumors and spleens, n= 6-8 mice per group. The measurements shown are expressed as the percentage of cells positive for CXCR3 multiplied by the median fluorescence intensity of CXCR3$^+$ cells. b) CXCR3 surface staining of CD8$^+$ αMUM3 T cells stimulated in vitro for 2 h with the different treatments. CXCL9 was used at 1 µg ml^{-1}, TNFα at 50 ng ml^{-1} and IL-12 at 2 ng ml^{-1}. The percentage of CXCR3$^+$ cells relative to untreated cells is shown in the graph.

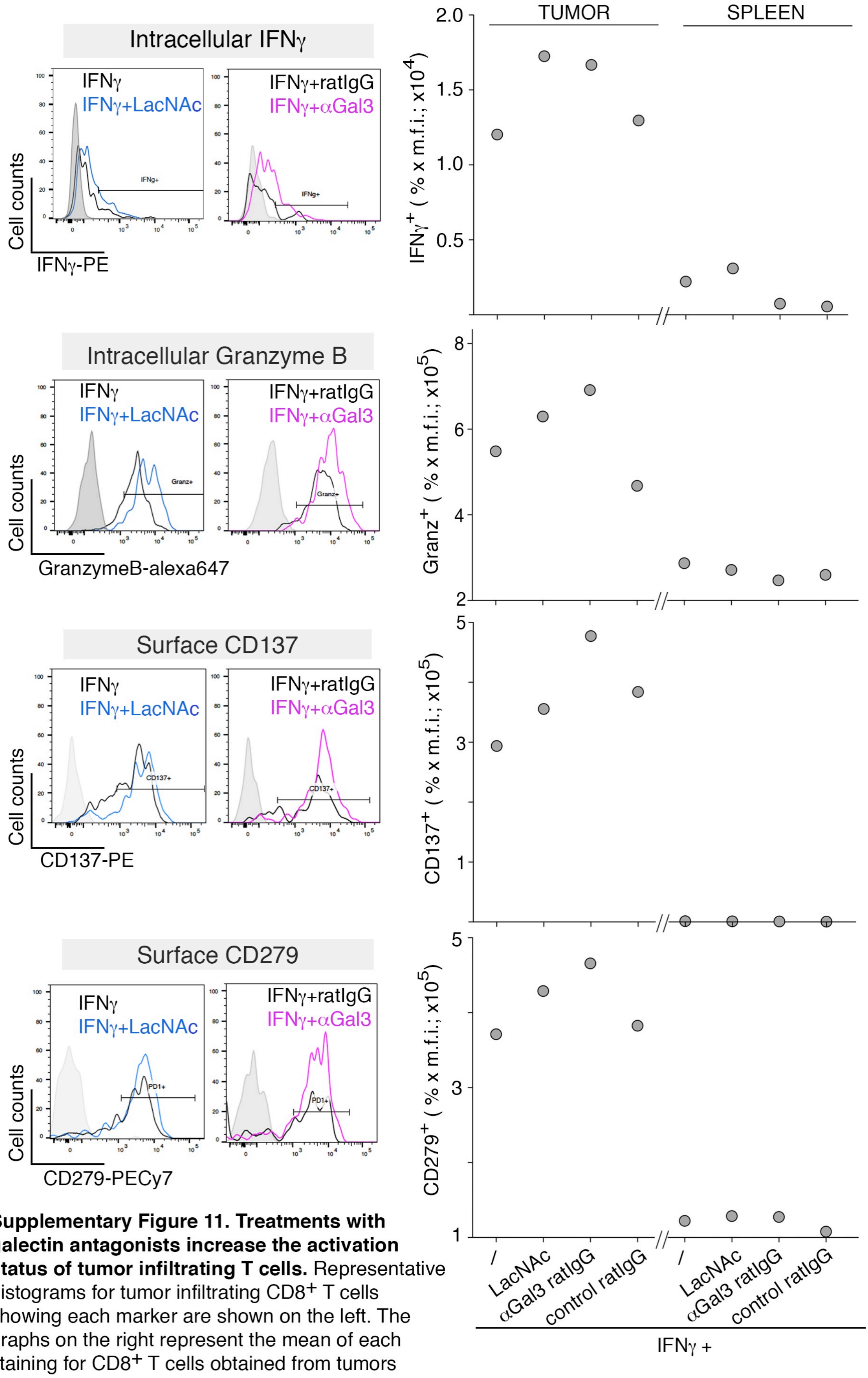

Supplementary Figure 11. Treatments with galectin antagonists increase the activation status of tumor infiltrating T cells. Representative histograms for tumor infiltrating CD8$^+$ T cells showing each marker are shown on the left. The graphs on the right represent the mean of each staining for CD8$^+$ T cells obtained from tumors and spleens. Data shown are expressed as the percentage of cells positive multiplied by the median fluorescence intensity (m.f.i.) of these positive cells for each staining, n= 6-8 mice per group.

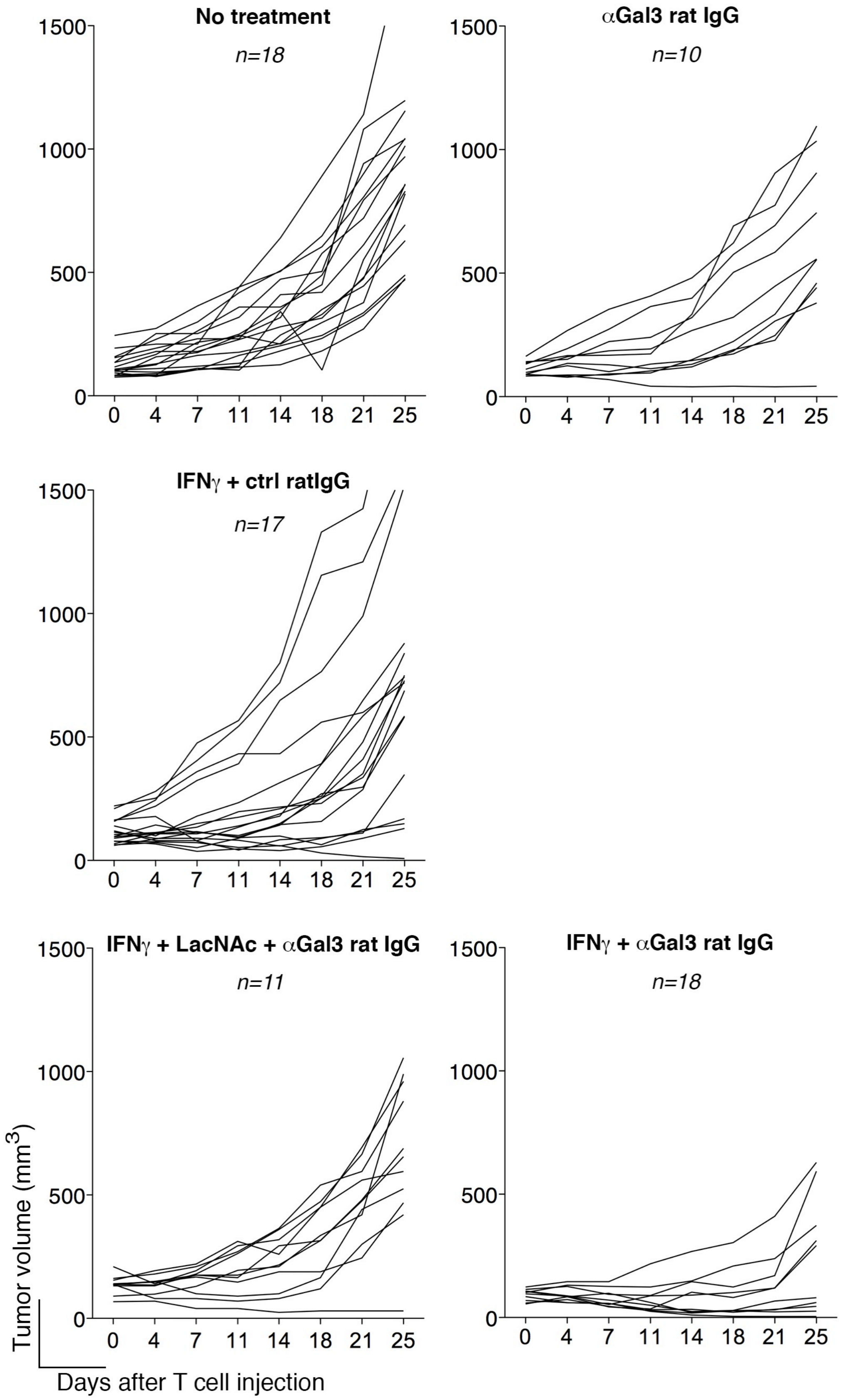

Supplementary Figure 12. Individual tumor growth for each mouse treated. The number of mice is shown below the treatment. The average tumor growth in shown in Figure 8b and 8c.
